# Consolidating Dispersed Knowledge About Citizen Science and Citizen Observatories: Experiences from the Four WeObserve Communities of Practice

**DOI:** 10.1007/s00267-026-02442-z

**Published:** 2026-04-25

**Authors:** Uta Wehn, Dilek Fraisl, Joan Masó Pau, Mohammad Gharesifard, Linda See, Gerid Hager, Jessica L Oliver, Tova Crystal, Raquel Ajates, Ane Bilbao, Eglė Butkevičienė, Carlo Andrea Biraghi, Jillian Campbell, Laurent Durieux, Eugenio Gervasini, Margaret Gold, Gitte Kragh, Andreas Matheus, Stephen Mac Feely, Lukas Mocek, Maina Muniafu, Lea Shanley, Ali Yousefi

**Affiliations:** 1 CWTS - Centre for Science & Technology Studies, Leiden University, Leiden, The Netherlands; 2 IHE Delft Institute for Water Education, Delft, The Netherlands; 3 International Institute for Applied Systems Analysis (IIASA), Laxenburg, Austria; 4 CREAF, Barcelona, Spain; 5 University of Groningen, Groningen, The Netherlands; 6 Australian Citizen Science Association Inc. (ACSA), Maleny, QLD Australia; 7 Consejo Superior de Investigaciones Científicas, Madrid, Spain; 8 Biotechnology Department, Delft University of Technology, Delft, The Netherlands; 9 Kaunas University of Technology, Kaunas, Lithuania; 10 Politecnico di Milano, Milano, Italy; 11 Secretariat of the Convention on Biological Diversity, UNEP, Montreal, Canada; 12 French National Research Institute for Sustainable Development, Paris, France; 13 European Commission, Joint Research Centre, Ispra, Italy; 14 Aarhus University, Aarhus, Denmark; 15 Secure Dimensions GmbH, Munich, Germany; 16 OECD, Boulogne Billancourt, France; 17 Sensor.Community, Stuttgart, Germany; 18 United States International University-Africa, Nairobi, Kenya; 19 International Computer Science Institute, Berkeley, CA USA; 20 Isfahan University of Technology, Isfahan, Iran

**Keywords:** Citizen science, Citizen observatory, Community of practice, Evaluation, Impact, Sustainable Development Goals

## Abstract

A strong Community of Practice (CoP) can be powerful in supporting people to share, generate, and disseminate knowledge. This study evaluates the use of the Communities of Practice (CoP) approach for effective knowledge consolidation in the field of citizen science. Our paper offers an analysis of four CoPs that were set up as part of the European-based 3-year WeObserve project, with distinct themes of (1) co-design & citizen engagement; (2) impact and value for governance; (3) interoperability and standards; and (4) the United Nations Sustainable Development Goals. Participation across the four CoPs fluctuated during their three-year life-time. Three key outcomes emerged from the CoPs. First, a joint identity and understanding were created within and across CoPs through the creation of an inception report by each CoP and through the creation of Citizen observatory (CO) vocabulary, which also served to differentiate such observatories from citizen science (CS) initiatives. Next, scientific papers and technical reports were cooperatively produced by CoP members that represent a synthesis of CoP members’ knowledge. Essential ingredients to the success of these CoPs also included extensive stakeholder engagement and the CoPs being steered by the underpinning values of the CS community. The impacts of the WeObserve CoPs range from the uptake of jointly produced publications, novel cooperative CS projects, new CoPs, joint grant proposals, and the integration of citizen science data into SDG monitoring. This evaluation highlights the diverse and transformative potential of CoPs for citizen science practice.

## Introduction

Citizen science (CS) has emerged as a way of involving individuals and communities in generating knowledge, whether through data collection or by identifying environmental problems that affect them (Shirk et al., 2012; Coulson et al., 2018; Ajates et al., 2020; Wehn, 2022). There are many examples of successful CS initiatives, such as eBird (Sullivan et al., 2014) and Galaxy Zoo (Clery, 2011), which have tapped into already established communities of motivated volunteers. Citizen observatories (COs) are a specific form of CS particularly found in the European context, which describe community-based environmental monitoring initiatives that make use of technology such as mobile phones or low-cost sensors to gather data needed to inform decision-making (Hager et al., 2021). Such observatories should therefore ideally have a two-way dialogue between the citizens and the organizations that need relevant information to support environmental policy decisions (Grainger, 2017). Along with the spread of citizen observatories and CS initiatives globally, the number of CS practitioners is growing. Yet many of these initiatives are struggling with challenges related to a lack of awareness of their existence, visibility, acceptability, and sustainability (Hager et al., 2021; Sauermann et al., 2020; Tolbert et al., 2025). Practice-based knowledge that successfully addresses these challenges is dispersed across multiple domains and various stakeholders in academia, the private and public sectors, and civil society, making it difficult to share successes and generate lessons learned across CS initiatives. There is both a lack of recognition and acceptance, by scientists, decision makers, as well as institutional procedures, that CS and COs can help fill data gaps, capture rapidly changing phenomena, define trends and support anticipatory science informing policy and create a space for dialogue (Schade et al., 2021, Wehn and Almomani, 2019). Coordination across COs holds promise to improve awareness of them across a range of stakeholders, increasing the acceptability, and fostering observatory participation and resource their sustainability (Liu et al., 2014; Nicolini et al., 2022). The WeObserve project was designed with the intent to address these three core challenges.

Specifically, WeObserve^1^, funded by the European Union as part of its Horizon 2020 program, aimed to improve the coordination between existing COs and other citizen-based environmental monitoring activities, in part, through Communities of Practice. The project explored Communities of Practice as one mechanism to address such challenges. A Community of Practice (CoP) is defined as a group of people that create and share knowledge on a specific topic, within a discipline or related to a particular skill (Lave and Wenger, 1991; Wenger et al., 2002; Verburg and Andriessen, 2006)^2^. Such a CoP can involve people within a single organization or across many geographically dispersed organizations. Additionally, a CoP can forge new partnerships between key stakeholders, advocate and contribute to knowledge consolidation at national, regional and international levels.^3^ According to Wenger et al. (2002), structural CoP elements are the domain knowledge (i.e. the specific set of focus issues), the community (people who care about the domain), and the practice (i.e. specific knowledge that the community develops, shares and maintains, and the shared practice of the community members that they are developing to be effective in their domain (e.g. frameworks, ideas, tools, styles, stories)). Key activities of CoPs consist of a) thematic knowledge co-creation, b) generating new solutions or agreeing on how to use existing ones, and c) knowledge sharing activities (Wenger et al., 2002).

Impacts of CoPs can be achieved on a large scale. For example, there are CoPs with documented impacts in the healthcare sector (Noar et al., 2023), in business (Senge et al., 2006) and in education (Every Learner Everywhere, 2023). The Community Activities of the Group on Earth Observations (GEO), a collaborative inter-governmental body sharing information and knowledge related to Earth Observation, also embody CoPs that cover a wide range of activities, including CS.

Within the WeObserve project, four CoPs were initiated and implemented to tackle the challenges of awareness, acceptability, and sustainability of COs and CS initiatives more generally. These include the Engage CoP, Impact CoP, Interop CoP and SDG CoP:

Engage CoP: Co-designing citizen observatories and engaging citizens: identify, define and share best practices related to engagement and co-design practices in CS and COs.

Impact CoP: Impact and value of citizen observatories for governance: create an inventory of methods, share best practices and provide guidance on CO impact assessment.

Interoperability CoP: Interoperability and standards for citizen observatories: demonstrate the interoperability of CS projects and COs, and to investigate the ways that relevant standards can be applied to data from CS and COs.

SDGs CoP: UN Sustainable Development Goals (SDGs) and COs: understand the opportunities and challenges for the use of data from CS/COs for SDG monitoring and implementation.

Each CoP was co-designed with CS practitioners (often members of past or present COs), researchers, and other interested stakeholders, and opened up to the broader community for anyone to participate (see Table 2 in the supplementary material). Membership of WeObserve CoPs drew from (primarily European) national, regional, and international CS associations and their working groups. Overall, CoP members were based in more than 40 countries, and much of the communication was digitally mediated. Additionally, a yearly face-to-face meeting (called CoP forum) was organized during conferences or as standalone events to strengthen social interactions between members. A problem-solving approach was applied to meet the objectives of each CoP (Lave and Wenger, 1991) through a combination of applied and research activities, i.e., jointly defining and analysing thematic issues and reviewing or developing approaches and solutions to these real-world problems in the practice of citizen science.

In this paper, we aim to evaluate the process of setting up and running the WeObserve CoPs, experiences participating in them, and the outcomes and impacts achieved thus far for knowledge consolidation in the field of CS. Using a tailored conceptual framework for evaluation and a case study approach, the methodology is described in detail in the next section. The results are then presented, followed by the impacts that have been realized beyond the lifetime of the WeObserve project. Finally, reflections on the findings, the challenges that were faced by the CoPs, and the limitations of the study are discussed.

## Methodology

The participatory evaluation of the four WeObserve CoPs with CoP members consisted of (i) developing the conceptual framework, (ii) designing and implementing the survey instrument, (iii) designing and implementing the post-project inquiry, and (iv) analyzing and interpreting the data. Steps (i) and (ii) were undertaken jointly by the CoP leads with CoP members. Steps (iii) and (iv) were undertaken by the CoP leads after WeObserve had finished.

### Developing a Conceptual Framework to Guide the Data Collection and Analysis

There is a substantial literature on CoPs (Brown and Duguid, 1991; Lave and Wenger, 1991; Hoadley, 2012; Kimble et al., 2008), yet there is a notable gap in available methods for the empirical evaluation of CoP processes, outputs and impacts (Meessen and Bertone, 2012). This is likely because the potential outcomes, such as innovation, learning and social capital, are difficult to measure. McKellar et al. (2014) reviewed 16 CoP evaluation frameworks. Of these, only nine were applied or tested in case studies. Moreover, seven of these were applied to CoPs within a single organization, and only two dealt with CoPs that included members from multiple institutions. Nonetheless, there are a number of frameworks that can be drawn from to evaluate the effectiveness of CoPs in supporting people to exchange, generate, and apply knowledge. To gain an understanding of the WeObserve CoP experiences in a consistent and resource-efficient manner, members of the WeObserve CoPs conducted a cross-comparison of relevant frameworks by Verburg and Andriessen (2006), Meessen and Bertone (2012), Wenger et al. (2011), and Gharesifard et al. (2019), assessing the strengths and weaknesses of each framework in terms of relevance and readiness for application in the WeObserve context. Concluding that none of the frameworks on its own was suitable and readily applicable for the evaluation of the WeObserve CoPs, the CoP members set out to develop a tailored conceptual framework, drawing heavily on Meessen and Bertone (2012) as well as on specific elements of the above-mentioned frameworks for evaluating the resources, processes, results, outcomes and impacts of the four WeObserve CoPs. The resulting WeObserve CoP framework featured three aspects, including inputs for establishing the CoPs, the process of how CoPs functioned, and CoP generated results, with each aspect having multiple elements and sub-elements (Table 1).

**Table 1:** An overview of the conceptual framework for evaluation of the WeObserve CoPs

Aspect	Element	Sub-element	Source
Inputs	Resources	Available resources ^c^[Resources provided by WeObserve and efforts by members]	Meessen and Bertone (2012); Gharesifard et al. (2019); Verburg and Andriessen (2006)
Process	Participation and facilitation dynamics	Goals and objectives ^c^[Main CoP objectives and members' goals]	
		members ^b^[Participants of the CoP]	
		Community activities, interactions and networking ^c^[Details of community activities, interactions and networking opportunities]	
		Facilitation ^c^[Reflection on the facilitation process]	
Results	Achievement of goals and objectives	Level of achievement of goals and objectives ^a^[The extent to which the CoP objectives and members' goals are achieved]	Wenger et al. (2011); Gharesifard et al. (2019)
	Individual/ organizational outcomes	Potential value: Knowledge capital ^a^[Activities and interactions can produce *“knowledge capita*l” whose value lies in its potential to be realized later]	Wenger et al. (2011), Verburg and Andriessen (2006)
		Applied value: Changes in practice ^a^[Examining applied value means identifying the ways practice has changed in the process of leveraging knowledge capital]	
	Emerging impacts	Emerging impacts ^c^[Impacts that may materialize over a longer period because of the CoPs]	Gharesifard et al. (2019)
	Future developments	Future developments ^a^[Points for improvement of the WeObserve CoP(s) in the future]	The authors

Relevant data sources for each sub-element:

^a^Primary data (survey; follow-up inquiry)

^b^Secondary data (CoP registration forms, minutes from CoP meetings, inception reports, etc.)

^c^Both primary and secondary data

### Designing a Survey Instrument to Explore Member Experiences

The next step was to design a survey instrument for online deployment to gain insights regarding the inputs, process and results aspects of the WeObserve CoPs, based on the conceptual framework (see Table 1 in supplementary materials). Two co-authors were involved in creating the initial draft of the survey (including 10 sections and 33 questions), designed in Survey Monkey and then circulated for feedback amongst the CoP members who attended a WeObserve CoP forum. Eleven CoP members provided feedback on the survey design that identified and proposed ideas for reducing structural and linguistic ambiguities, as well as increasing clarity with the inclusion of descriptive examples and alternative multiple-choice options. The final version of the survey included 10 sections and a mixture of 29 open-ended and closed questions.

The invitation to participate in the survey (2020 survey hereafter) was sent to 280 WeObserve CoP members, and was open between 20 November and 11 December 2020. Out of those 280, 143 people were participants (members who attended at least 2 CoP meetings). The remaining members are considered observers. There were 13 instances of people who clicked on the survey link, but did not respond to any questions.^4^ Twent-eight people responded to the survey; twenty-one respondents completed the full survey, and seven completed parts of the survey.^5^

The majority of the questions were in closed form; however, almost all questions included an open text box that the respondents could use to provide elaborations and concrete examples for their answers.

### Post-Project Inquiry Among Cop Members

Three years after the end of the WeObserve project and the respective CoPs’ activities (January 2024), a follow-up inquiry with open questions (sent via email) was conducted with the members of all four CoPs. We sought to understand how participation in a WeObserve CoP supported new perspectives and new ways of doing things; encouraged the use of outputs of the CoPs; and fostered relationships. The instrument included the following questions:

- In what ways have the WeObserve CoP(s) provided you with new perspectives and/or new ways of doing things?
- What specific outputs or methods generated in the WeObserve CoP(s) have you used/benefited from?
- If you have, how have you used or built on these outputs or methods (e.g., in follow-up projects)?
- How have you leveraged the relationships/network(s) created via the WeObserve CoP(s)?

In total, nine CoP members responded with detailed answers to these open questions. The response rate was likely low due to the CoP activities ending roughly about three years previously, around March 2021. It is also important to note that due to the nature of self-evaluation in this instrument, there is a potential for attribution bias. A summary of how the evaluation methods accompanied the CoPs activities is provided in Table 2.

**Table 2:** Overview of the timing of CoPs activities and the implementation of evaluation methods

	2017	2018	2019	2020	2021	2024
**CoPs activities**	Q3: WeObserve project start	Launch of the CoPs Forum #1 (Q2, Geneva) & #2 (Q4, Venice)	Forum #3 (Q2, Vienna) & #4 (Q4, Barcelona)	Forum #5 (Q4, online)	Forum #6 (Q1, online) CoPs concluded WeObserve project end	
**Evaluation methods implementation**				Q3 Survey co-design Q4 Survey implementation (sent to all CoP members)		Q1 Post project inquiry instrument – design & dissemination among all CoP members

### Data and Data Analysis

The primary data set was created from responses to the 2020 survey, which was administered in the final year of the 3-year WeObserve project. The survey was organized in Excel and analyzed in line with the conceptual framework, focusing on CoPs Inputs, CoPs Process, and CoPs results. The quantitative responses to the survey instrument were analyzed and triangulated with a thematic analysis of the open-ended responses. Further empirical data obtained from the CoP members via the post-project inquiry were collated per item in Word and analyzed thematically and per CoP (as applicable) to identify further insights and to compare advances since the 2020 survey. Selected aspects were complemented by information obtained via desk research (e.g., status of citations of the scientific papers and reports produced by the CoPs). Secondary data sources (i.e., co-created CoP meeting minutes from 71 CoPs meetings, four CoP Inception Reports that were co-created with CoP members during the process of setting up the CoPs (see section 3.2), and the minutes from six CoPs fora spanning 17 days of F2F meetings in total), were drawn upon and integrated in the overarching assessment of the CoPs according to the conceptual elements (CoP inputs, CoP process, CoP results). Overall, the analysis was guided by the elements of the conceptual framework. For specific elements from the conceptual framework, results from the primary analysis were reflected upon and juxtaposed with insights from the secondary data.

## Results

This section presents the results of our analysis, namely an examination of the WeObserve CoP inputs, CoP processes, and CoP results. Each subsection includes an analysis of data from surveys described above.

### CoP Inputs

**Fig. 1:**
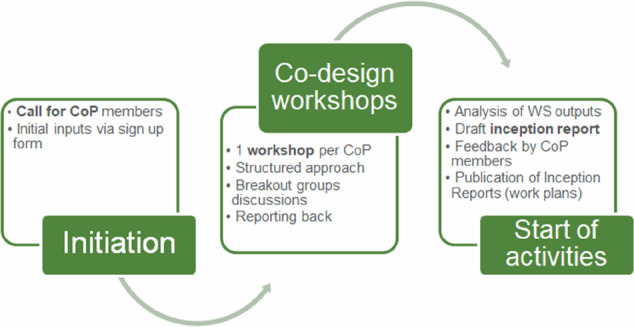
Overview of the WeObserve co-design process

The operations of the WeObserve CoPs were funded by WeObserve project resources, which included preparation, initiation, co-design and operational support, annual face-to-face meetings and specialized workshops, preparatory activities for the launch of each CoP (see illustration in Fig. 1), web presence, and administrative support. These were used to produce Terms of Reference 6 which provided clarity on WeObserve CoP participation for future CoP members. Moreover, preparations for the launch of the WeObserve CoPs included the following:

- Promotion of the launch of the CoPs and a Call for CoP members on the WeObserve website and social media channels
- Preparation and processing of registration forms to obtain CoP members’ GDPR consent, insights on the expectations and potential contributions.
- Active member recruitment during key events leading to the CoP launch
- Preparation of the face-to-face CoP launch event with detailed session design, slides and supporting material (see Wehn et al., 2019)

**Fig. 2:**
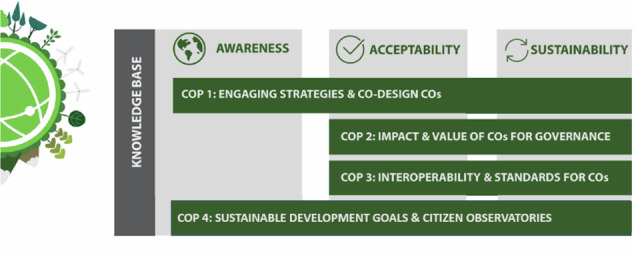
The challenges that each CoP addressed

All CoPs aimed to address one or more of the key challenges that the WeObserve project identified as barriers to mainstreaming CS, namely awareness, acceptability, and sustainability (see Fig. 2).

The first aspect of the conceptual framework refers to the resources of the CoPs both from the WeObserve project and from the members themselves. Members dedicated their time to the CoPs which also often meant a financial investment as well. Figure 1 in supplementary material shows the many ways in which members financially invested in the CoPs. From time and resources to travel expenses, all respondents indicated some sort of financial contribution during their participation in the CoP. Figure 2 in supplementary material shows how much of their time members dedicated to the CoP activities (which ranged between 1 to 8 hours per month).

### CoPs Process

#### Launch of the CoPs

All four CoPs were launched in 2018 during face-to-face events, with an average of 20 members per session, using a consistent, co-design process (Fig. 1) that was aimed at obtaining insights on the structural elements of each CoP, namely domain, community and practice (Wenger et al., 2002). The overarching broad topics were pre-selected while the ideas contributed by members (i.e. a form of input) during interactive launch sessions laid the structural elements for each CoP (Table 3). Each CoP chair drafted an inception report specifying the target audience, needs, activities, and objectives and a detailed work plan (as detailed in Wehn and Velzeboer, 2018a, 2018b; CREAF et al. (2018); Fraisl and Wehn, 2019). These inception reports were shared for comments with the respective CoP members and subsequently finalized. They defined a clear path for the CoPs and were used as a reference point to keep the CoPs relevant, engaging, and focused for their members. The WeObserve project had a geographic focus on Europe which is reflected in the CoPs; however, they remained open to anyone. While the target participants varied slightly per CoP, the stakeholders encompassed Civil Society Organisations, research & academia and citizen science working groups.

**Table 3:** Overview of the WeObserve CoPs structural elements

CoP	Domain (i.e., thematic focus)	Community (i.e., members)	Practice
**Engage CoP**	(1) Strategic engagement practices - efforts to engage different demographic and geographic target groups based on understanding incentives and barriers (2) Co-design methods, including co-design conditions, versions and tools	CS practitioners from universities and research institutions, UNESCO, GEOScience Australia, GLOBE program, NASA, JRC, GPSDD, local governments, NGOs, private companies	Producing effective CO knowledge resources on co-design and citizen engagement.
**Impact CoP**	(1) Governance dynamics: understanding existing decision-making structures and the role of COs (and their data) in changing these to address societal challenges (2) Impact stories: capturing and demonstrating the impact and value of COs for governance	CS practitioners from universities and research institutions, representatives from the European Commission, GBIF secretariat members, local governments, NGOs and private companies.	Generating effective CO knowledge resources on the impact and value of COs for governance.
**Interop CoP**	(1) The use of OGC standards (e.g., Sensor Web Enablement for CS SWE4CS) to support data integration among CS projects, and with other sources, esp. authoritative data; (2) The integration of CS projects/campaigns in a Single Sign-On system (SSO) federation; (3) The relationships between OGC standards and data and metadata standards currently used by CS projects.	CS practitioners from universities and research institutions, representatives from UNITAR, the World Meteorological Organization, the European Commission and private companies	Demonstrating the interoperability of CS projects and the way OGC standards can be applied to CS.
**SDG CoP**	(1) UN Sustainable Development Goals (SDGs) and (2) CS/Citizen Observatories	UN custodian or partner agency representatives (UN Environment, WMO, UNESCO, etc.), statisticians, policymakers at the EU level and broader, CS practitioners, academics, researchers, citizen-generated data specialists, etc., worldwide.	Exploring and demonstrating the value of CS data for SDG monitoring.

#### Participation and Facilitation Dynamics

All CoPs had *“founding members”* who were present from the start of the CoPs, either through initial registration and/or physical presence at the launch workshop. During the lifetime of the WeObserve CoPs, the CoP members met frequently via monthly online meeting calls and annual multi-day interactive events, referred to as CoPs fora. The interactive CoP-focused fora were often held in conjunction with broader academic and industry events to create synergies and recruit additional CoP members. The CoP fora were used as opportunities to cooperatively reflect on the interim outcomes, plan the next steps, and to foster networking and relationship building within and across the CoPs. Prior to the COVID-19 pandemic, events were offered as hybrid events to allow for the participation of CoP members outside Europe. In addition to these plenary meetings, the CoP chairs met as needed to maintain alignment, as well as to discuss events and activities concerning all CoPs.

The WeObserve Terms of Reference were created to explicate standards for maintaining a high level of professionalism and consistency within the CoPs. Registration to join a CoP involved signing an EU General Data Protection Regulation (GDPR) compliant agreement. Along with the inception report, each CoP produced status updates twice a year and concluding reports. For all monthly meetings, an agenda was made by the respective CoP chair, and minutes were kept during every CoP online meeting via a shared online document, including attendance. All CoP members were encouraged to co-create the minutes in real-time, and a designated WeObserve staff member also helped to take minutes during meetings.

Regular meetings were usually structured with time for guest speakers, information exchange about relevant events, and collaboration opportunities. Guest speakers sometimes represented local CS initiatives, key actors outside the CS community (e.g., national statistical agencies), or CoP members. During CoP monthly online meetings, challenges initially included fostering interactions among members and eliciting contributions from them. This was typically overcome through several actions. One such action was creating task forces composed of dedicated members that would drive the tasks forward, such as producing templates, glossaries, papers, conference session abstracts, and testing of tools and approaches. Another action was enabling more constructive discussion during the regular CoP online meetings. These regular agenda items helped ensure that discussions were relevant and beneficial for those participating, encouraging individuals’ commitment to the CoP.

The positive answers in the 2020 survey reflect the usefulness of the above structure for at least some of the CoPs participants (i.e., among those who participated in the survey). The facilitation of interactions during the monthly CoP online meetings was rated predominantly “*very good*” or “*good*” (Fig. 3 in supplementary material). The respondents indicated that the regular CoP online meetings were very well-organized, the CoP leads were very active, and the discussions in the regular CoP online meetings were moderated effectively. Respondents perceived the regular meetings, presentations on a particular topic, connections created (or strengthened) among members, synergies with other working groups^7^ and continuous updates on events, papers, etc. as helpful for achieving the CoPs’ objectives. These findings also resonate with results from the analysis of the secondary data, with appreciative comments made to this effect by CoP members during CoP meetings and fora, either spontaneously or solicited (e.g., during end-of-meeting reflections).

Over the three-year lifetimes of each CoP, 280 individuals registered; 104 people registered for one CoP, while 176 people registered for two or more. Of those registered, almost 50% (143 participants) were actively engaged in the CoPs (see Table 2 in supplementary material). To gauge the level of engagement within the CoPs, the term “*participant*” is used to refer to individuals who attended at least two CoP online meetings. Across all four CoPs, 33 participants attended 5 or more online meetings, and 25 participants attended 10 or more. Many people attended more than this, while others joined once. The members were mostly from academic and research organizations, but also represented the public sector, NGOs, and occasionally the private sector. The SDG CoP, in particular, was successful in reaching out to other communities, including the National Statistical Offices (NSOs), UN bodies, policymakers and the broader data and statistics communities. The Interop CoP also connected with the Group on Earth Observations (GEO), as well as the standardization bodies (such as the OGC).

### CoPs Results

Five conceptual elements were used to evaluate the results of the CoPs (see Table 1), namely (i) Level of achievement of goals and objectives; (ii) Potential value: Knowledge capital; (iii) Applied value: Changes in practice; (iv) Emerging impacts (v) Future developments. A summary of the CoPs' objectives, activities and results is presented in Table 4.

**Table 4:** A summary of the WeObserve CoPs' objectives, activities and results

CoP	Objectives	Activities	Results/Impacts
**Engage CoP**	—To identify and define strategic engagement practices and CO co-design aspects and elements. —To capture lessons learned (from success and failure) from the implementation of strategic engagement practices and CO co-design versions and tools in differing conditions. —To share these methods and lessons learned via the WeObserve Cookbook on Citizen Observatories and via other means.	Co-create a glossary of key terms relevant to co-design and engagement in CS and COs Jointly capture, analyze and synthesize lessons learned across projects	—Key terms for WeObserve glossary —Inputs for WeObserve Cookbook on co-design and engagement practices —Joint H2020 Green Deal proposal by core CoP members —2 policy briefs —Report on findings on bottlenecks and barriers —Template for inventorizing engagement practices and CO co-design conditions, versions and tools
**Impact CoP**	—To provide an inventory of “tried and tested” methods for capturing the impacts of COs on governance —To capture CO impact stories/examples of best practice from citizens, the public sector and policy perspectives —To provide guidance on CO impact assessment for the CoP members and beyond	Develop a practical tool for capturing the impacts of CS Run storytelling workshop Paper writing meetings Set up a template to capture impact stories	—CS Impact Storytelling Approach (CSISTA), now used by the CitiObs project (HEU 2023-2026) —Overview of state-of-the-art impact assessment for CS & best practice principles —Informing the work of the current Horizon Europe project Urban ReLeaf —ECSA Working Group on Impact Assessment —Scientific publications (see Table 4 in the supplementary material)
**Interop CoP**	—Successfully demonstrate how OGC standards (e.g., SWE) are applicable to CS, document available supporting tools, identify the challenges of using OGC SWE standards (or Internet of Things equivalent solutions) within current CS projects, and propose a way forward. —Determine the security considerations and the available tools to support an SSO federation that helps users participate in several projects by using a single user account. —Assess the possible relationships of OGC standards (e.g., SensorML) with other existing standards in the field (e.g., Public Participation in Scientific Research (PPSR) - Core, the ontology developed by the COST Action on CS, and the CS Definition Service (CS-DS) developed in the NextGEOSS project). —Satisfy the necessary requirements to integrate CS into GEOSS by using OGC standards.	Interoperability experiments testing data sharing and data integration Writing technical recommendations and engineering reports.	—OGC best practice document for using sensor standards for CS —OGC STAplus standard that extends SensorThings API —CitiObs uses STAplus for exposing data on air quality for different providers and for integrated observations —The more4nature project (HEU 2024-2027) uses STAplus as a repository of harmonized data and for the alerting system —The Lisbon Declaration recognizes the need to integrate citizen-generated data with official sources. —Scientific publications (see Table 4 in the supplementary material)
**SDG CoP**	—Understand the opportunities for COs/CS in SDG monitoring and implementation, and integrate these into the WeObserve knowledge base/community and learn from existing partnerships (i.e., projects and custodian agencies, national statistical offices)	Compile a list of minimum data requirements/standards for CS/OS Mapping exercise of CO/CS projects and the SDG indicators	—Scientific publications (see Table 4 in the supplementary material) —Contribution to the SDG indicator 14.1.1b methodology development led by UNEP —Integration of CS data into official marine litter statistics in Ghana —Media coverage of the use of CS data in SDG monitoring, including an article in Forbes, a Nature editorial, and many others.

#### Achievement of Goals and Expectations

First, a joint identity and understanding were created within and across the CoPs, resulting in the production of a joint glossary (across the CoPs). The 47 entries in the glossary define key terms and concepts, based on key references and arrived at via discussions within and across the CoPs; among other things, this also serves to differentiate citizen observatories from CS initiatives. The glossary is still available online and continues to serve the global community of practitioners^8^.

One of the overarching purposes of the WeObserve CoPs (and also an expectation by the CoP members individually) was the consolidation of knowledge in scientific articles and reports. In total, the WeObserve CoPs cooperatively produced 15 publications, including academic articles and technical reports, that represent a synthesis of CoP members’ knowledge (Table 4 in the supplementary material). The information from these publications has been extensively applied in various research, advocacy, and educational activities and contexts by former CoP members and broader stakeholders.

Figure 4 in the supplementary material shows the respondents’ impression of the degree to which the CoP objectives had been achieved. Some respondents from each CoP indicated that such achievements had been reached, with this sentiment being strongest in the Impact CoP, where 12 (of 22) respondents indicated “to a large extent”. No one answered “not at all”, although the second highest category was “not able to judge”: Engage CoP 8 (of 23) respondents, Impact CoP 7 (of 22) respondents, Interop CoP 11 (of 19) respondents, SDG CoP 11 (of 24) respondents. One respondent who self-identified as an observer shared that they were “n*ot aware of particular objectives, but after reading them, I have given my impression of* *success*”. The objectives for the CoPs focused on creating value for the members. In this case, the achievements of the objectives of the CoPs were observable for members even when not working specifically toward them.

Each CoP member had their own expectations about their participation in the respective CoP (Fig. 5 in supplementary material). The diagram highlights common expectations across all four CoPs, such as networking with relevant practitioners and/or other networks, which were frequently mentioned. However, some expectations were more prominent in certain CoPs. For example, members of the Interop CoP showed a strong interest in helping to improve CS activities in their own projects and making CS expertise available to others. These expectations were less emphasized among the SDG CoP members. Access to new knowledge, tools and methods, as well as the production of joint publications, were most prominent among the Engage CoP, Impact CoP and the SDG CoP. The respondents to the 2020 survey were also invited to reflect on the realization of their initial expectations of participating in the CoPs. Among those who answered this question, the majority indicated that their expectations had been met to a large extent (Fig. 6 in supplementary material). Two respondents who reported that their expectations had not been met, both shared that this was largely due to their own time limitations for participating.

There is further evidence of many of the CoP expectations and objectives being met. Across all four CoPs, members participated in at least 41 different events, both online and in-person, that directly related to the aims of the given CoP. Some CoPs, such as the SDG CoP, were proactive in sharing their work. This speaks to two of the expectations from participating in the CoPs: *access to new knowledge* and *dissemination opportunities*. Members worked on multiple projects together during the lifetime of the CoPs, and significant updates and project events were often discussed during the monthly online meetings.

CoP members also expressed a sentiment of having received access to new resources (see Fig. 7 in supplementary material) through their involvement in the CoP (Engage CoP: 12 (of 20) respondents Impact CoP: 11 (of 18) respondents; Interop CoP: 5 (of 16); SDG CoP: 11 (of 20) respondents) The templates produced in the Impact CoP were mentioned in the 2020 survey as a useful resource that has applications beyond the CoP activities. For the other three CoPs, the majority of the respondents report a moderate increase in access to (new) resources. Learning about tools/methods from other disciplines, links to conferences, and access to the SensorThings API were among the explicit examples mentioned. Much of this access was made possible through the online meetings and the above-mentioned structure to involve external speakers, internal exchanges, and a permanent, dedicated virtual space for resources to be linked and shared.

#### Leveraging the Relationships Created Via the WeObserve CoPs

Via the 280 individuals who registered to be members of the CoPs, 211 unique organizations were represented. While not all 280 individuals became active members of the CoPs (Table 2 in supplementary material), these individuals had access to the online meetings and resources shared by the CoP. This large number of organizations/institutions that to some extent shared common goals and interests allowed for new collaborations and networks for members. Figure 8 in the supplementary material shows the respondents’ answers to the corresponding survey question on the creation of collaboration opportunities. In the case of the Engage CoP, Impact and SDG CoPs, the number of *(strongly) agree* responses was higher than neutral answers (Engage CoP 6 (of 19) respondents, Impact CoP 7 (of 18) respondents, Interop CoP 3 (of 17) respondents, and SDG CoP 7 (of 24) respondents) with multiple other respondents from the Engage, Impact, and SDG CoPs indicating “agree”. Opportunities for working with new people in writing collaborative papers and project proposals were two of the explicit examples mentioned by the respondents. Furthermore, collaboration opportunities have led to lasting networks in some cases.

In the 2020 survey, the connections made from the CoPs and opportunities that arose because of these networks are more pronounced, with 15 responses indicating that they utilized the relationships and networks made in the CoPs *to a “large extent”* and another 13 responses indicating they did so *to a moderate extent* (Fig. 9 in supplementary material). Two respondents highlighted that the connections they made led to joint proposal writing. Some of the relationships formed and fostered within the WeObserve CoPs were instrumental in the success of subsequent projects. The networks of the Interop CoP intersected with other groups like GEO Citizen Science and OGC Citizen Science Standard Working Group, leading to cross-fertilization and benefits for these groups that are continuing.

Participation in WeObserve CoPs has also led to various concrete career opportunities for the members, including invitations to give lectures, write blog posts, and serve as expert reviewers for European Commission proposals.

Despite the positive ways in which relationships formed and were leveraged, one respondent also highlighted some key ways in which networks were stymied since “*learning about the group and what you might get involved in seems tricky as examples of activities weren’t really publicly clear*”. This sentiment was echoed by another respondent who suggested that the CoPs should “*search for methods and opportunities to engage a wider range of stakeholders*”.

#### New Perspectives and New Ways of Doing Things

In the 2020 survey, respondents reflected on the effect of the CoPs on the innovation of their practice (Fig. 10 in supplementary material). A majority of respondents indicated that innovation in their practice had been positively impacted to a large or moderate extent (39 responses out of 73). A member of the Impact CoP identified storytelling and impact stories as “*totally new approaches*” for capturing the impacts of CS. The importance of storytelling as a tool was also explicitly mentioned during the data collection (post-project inquiry) in 2024. Respondents also indicated that there have been far-reaching impacts of the CoPs on CS. These impacts are best categorized as enrichment in the *understanding* of CS and enrichment in the *application* of CS.

According to CoP members (2020 survey), the CoPs advanced the application of CS and citizen observatories by improving approaches to environmental research. For example, the methodological integration of local knowledge with scientific inquiry, fostered by these collaborative and interdisciplinary CoPs, led some CoP members to adopt a more community-driven research methodology in their practice. This shift emphasizes community engagement as a core element rather than a peripheral aspect of their CS practice. Furthermore, for certain members, this experience enriched their academic pursuits and teaching methods, reinforcing their dedication to integrating CS into education. Within the Interop CoP, a novel method of encoding and sharing citizen-generated data emerged (a generic model independent of thematic focus) and is now recognized as best practice (Matheus, 2022) and later as an OGC international standard (Matheus, 2023). This shift in perspective among CoP members facilitated cross-domain data communication, marking a significant advancement in collaborative data sharing.

#### New CoPs Based on the WeObserve CoPs Approach

The WeObserve CoPs have also continuously received interest for the way they were set up (using a specific co-design approach, see Wehn et al., 2019) and run using clear structures. Subsequently, additional CoPs have been set up (some by the WeObserve CoP chairs, others by CoP members) based on the WeObserve approach of having targeted participation, results, and impacts (Table 3 in supplementary material). The Citizen Science & Open Science CoP (CS&OS CoP) is a salient example, which was set up in 2020 and is currently the longest-running community of practice resulting from WeObserve.

In their reflections from the post-project inquiry, several CoP members expressed their explicit appreciation for the WeObserve approach and that they had learned hands-on how to create a CoP or Working Group, discuss shared objectives and keep the momentum in a community of practice. For example, the Urban ReLeaf Horizon Europe project started a Community of Practice and associated working groups in 2023, adopting practices from the WeObserve CoPs to implement an effective knowledge creation and sharing strategy. Additionally, the recent EU-funded project, CROPS^9^, will establish “*transnational communities*” in alignment with the EU missions, leveraging the learnings from the WeObserve CoPs to establish and run these communities. Several broader CoPs have subsequently followed the WeObserve project approach for creating and maintaining CoPs (Table 2 in supplementary material).

## Discussion

A Community of Practice is more than codified knowledge (e.g., a website, database or best practices), namely “*a group of people who interact, learn together, build relationships, and in the process develop a sense of belonging and mutual commitment”* (Wenger et al., 2002, p.34). WeObserve extended to this practical level and applied these core concepts through the co-design approach for the CoPs.

The four CoPs varied significantly in the amount of tangible outputs. As an example, the SDG CoP produced 10 scientific publications, whereas the Engage CoP did not publish any articles. The main reason for these differences relates to the *input* aspect of the conceptual framework, specifically the sub-element of available resources from the CoP members. The SDG CoP was led by a doctoral candidate whose research directly related to the CoP, while the Interop CoP was led by a full-time researcher and the Engage CoP and the Impact CoP were both led by the same full-time researcher. Therefore, the number of official hours that the SDG CoP Chair was able to invest in the CoP was significantly higher than the other CoP leads who were supporting the CoPs as part of the WeObserve project alongside their larger portfolio of projects. This being the major differing factor in the CoPs points to the importance of CoP leadership and individual capacity (mainly available time) in driving the accomplishment of activities in the WeObserve CoPs. One SDGs CoP member perceived the WeObserve CoPs as being run somewhat “top down”. While this was valued for driving towards the success in the outputs, especially by the SDG CoP, there was also a desire to have a more community-driven, bottom-up approach. According to Lester & Kezar (2017), an appointed CoP leader is known to embody the “philosophy and values of the community” (p. 29) and plays a critical role in coordinating activities, recruiting members, and mediation. This is in line with the role the WeObserve CoP leaders took on. Muller (2006) explores the creation of organic CoP leaders who emerge as such due to their high level of participation and action within the CoP, finding that leadership figures in a CoP can curb issues with “task coordination and of work coherence” (p. 385). While this is not how the WeObserve CoP leaders came to be, they did serve these functions and were highly engaged in the CoP. The WeObserve CoPs did not face significant issues regarding the coordination of meetings or work. From the analysis of the 2020 survey and the secondary data, it is evident CoP members perceived that the CoPs were “very well organized”, that discussions were led “in [an] effective way”, and that “the regular meetings with presentations on particular topic[s] as well as continuous update[s] on events, papers, etc. was very helpful and helped to achieve objectives”. Many of these aspects are due to establishing leadership from the beginning, and these activities were funded by the WeObserve project. Wenger (1998) talks about a “diverse and distributed” internal leadership (p. 7) that can be nurtured during the lifetime of a CoP. While the WeObserve CoPs did have de facto leadership from those members who engaged more intensely with the activities, perhaps a more concerted effort to create distributed leadership could even out top-down approaches in CoPs with strong initial leadership and help progress CoPs where the leadership is already stretched rather thin. Arguably, there is no “one size fits all” approach to CoPs in terms of leadership style, level of formality and structure.

One of the key aspects in the literature on CoPs is that a CoP should exist as long as it creates value for its members (Wenger et al., 2011; McKellar et al., 2014). This was ensured in the WeObserve CoPs through the co-design, meaning the respective thematic foci of the CoPs were guaranteed to be relevant for their target audience, since it was the target audience who defined the specific scope and detailed focus. The practice documented and evaluated in this paper affirms this key guiding principle for CoPs. Through the co-design process, CoPs are highly likely to identify one or more topics of relevance at a point in time. However, CS represents a dynamically developing field of research and practice, and this means that CoPs need to be able to evolve to remain worthwhile for their members. This includes ending a CoP when the objectives have been achieved, and the maximum value for members has been reached. At the end of the WeObserve project, a discussion was held on the continuation of the CoPs. All four CoPs agreed that there was interest in continuing, and plans were made to try to secure funding and/or embed the CoP in other projects. This was successfully done, as in the case of the EU-funded CitiObs and Urban Releaf projects (see supplementary material) and via the Working Group on Impact Assessment of the European Citizen Science Association, started in 2024, which convenes many of the original WeObserve Impact CoP members and focuses on.

Versatility proved important for the CoPs in another way when the COVID-19 pandemic hit. The online nature of the regular meetings made the CoPs quite resilient to the sudden pandemic-induced societal changes. Nevertheless, the pandemic posed challenges to the CoP interactions that previously had taken place during the various forms of in-person fora and face-to-face meetings back-to-back with other conferences and events and which had played a critical role in strengthening the CoPs. While losing these interactions was difficult, the CoPs made a concerted effort not to lose this time for collaboration by changing the in-person meetings to be hosted online. Nevertheless, the in-person meetings that kicked off the CoPs were crucial for establishing a strong foundation in both ideas and relationships, and without these, the CoPs would likely not have endured as well during the pandemic.

A critical takeaway from the WeObserve CoPs is the “need to practice in CoPs what we preach more generally in CS” and focus on how to shape activities to engage stakeholders, help them overcome barriers to participation and create value in the joint learning derived from their communication and collaboration. There is room to identify ways and opportunities to engage a wider range of stakeholders in the CoPs: the WeObserve CoPs were populated primarily by academics and researchers, with much lower representation of especially civil society (organizations or individual citizens) and public and private sector organizations. This more homogeneous make-up may also explain why the functioning of the CoPs was widely viewed as successful, since academics and researchers are typically used to online workspaces with expertise in written work. Also, a significant challenge found in the WeObserve CoPs was the inability of some members to justify participation in the CoP as part of their work. Furthermore, many stakeholders are involved in Working Groups, Communities of Practice, Action Groups and more. Such activities involving volunteers are on top of existing professional obligations and commitments. Not surprisingly, many are experiencing a certain fatigue from being involved in “yet another” CoP or working group.

One important and tangible solution to overcome these barriers is funding. The WeObserve CoPs had a budget available through the WeObserve project for the CoP chairs as well as participants; this was a critical asset that enabled structured yet flexible organisation of the CoPs while also allowing members to travel and have (some of their) accommodation costs covered. Another way to address barriers to participation is by ensuring there are incentives for participation in the CoPs. Example incentives can include support for organising CoPs, as well as knowledge sharing and academic opportunities. Through the WeObserve CoP co-design approach, the incentives and benefits of the CoP are more likely to be relevant for the members, ensuring that they encourage and justify participation. The tools put in place to incentivize and mitigate any further fatigue were diverse. One was the adherence to a common set of rules that protects the intellectual property of the members and the privacy of their discussions. Another was ensuring that meetings and actions happen promptly throughout the process. Additionally, the WeObserve CoPs were run with a goal of accelerating knowledge consolidation through the demonstration of ideas and adapted solutions. Participation was also incentivized by fostering the sharing of experiences and knowledge across CoPs over time. The conditions created by the WeObserve project for the existence of meaningful CoPs in terms of financial resources, project tasks (for the CoP chairs) and deliverables (e.g., CoP ToRs and progress reports) were deliberate, reflecting evidence-based project design drawing on earlier CoP experiences and insights into participant incentives (e.g., Wehn et al., 2016). However, in view of continued budget cuts for research and innovation, creating such conditions is likely to become more challenging. This requires smart and strategic (re)use of scarce(r) financial resources and proven approaches.

The overarching aim of our study was to evaluate the WeObserve CoPs, which had been set up as part of the larger WeObserve project and its efforts to promote visibility, acceptability and sustainability of citizen science and citizen observatories. The findings of our study are tentatively positive with respect to the effectiveness of CoPs for overcoming knowledge dispersion among CS and CO practitioners; it goes beyond the scope of our study to assess the extent and attribution of the CoPs to improving the visibility, acceptability and sustainability of CS. Also, it is important to bear in mind that this study relied on a small sample of potential respondents from the overall population (280 CoP members) who, arguably, may also represent the most active CoP members (and most aware of the reliance of the evaluation on their response). At the time, their responses may suffer from a certain recall bias (either positively or negatively). Complementary to the responses to the survey and inquiry instruments, the tangible CoPs outputs (such as reports and publications) and their uptake were also assessed independently.

The tailored conceptual framework for the evaluation of the WeObserve CoPs helped to frame our evaluation effort while keeping it focused and manageable, especially its operationalization via a survey instrument to collect relevant information. At the same time, the survey instrument received a few critical comments from the respondents, pointing out that a small number of questions in the instrument could benefit from slight rephrasing. The question on how participation in the WeObserve CoP(s) transformed *how* participants learn could be accompanied by examples, whereas the question about the content of new knowledge and insights (i.e., new ways of doing things, new perspectives and/or new concepts in the field of CS) was perceived by some respondents to overlap with the question about learning processes. The post-project inquiry step was added to the initial study design due to COVID-related delays in completing the data analysis. Nevertheless, it generated suitable and useful additional insights on outcomes and impacts that had emerged since the termination of the CoPs. For future studies, this can provide a suitable approach for longitudinal studies of CoPs. Also, reflecting on the application of the framework in this study, it can be argued that two additional sub-elements in the *Process* aspect may be useful to include, namely the process of setting up the CoPs and the CoP leadership capacity and style (top-down vs. bottom-up).

## Conclusions

The WeObserve CoPs had the purpose of consolidating practice-based knowledge about CS and citizen observatories dispersed among practitioners. They supported practitioners in making connections with each other and expanding knowledge on COs and CS. This study offers an understanding of the influence that Communities of Practice can have in supporting and advancing the field of CS.

Three key outcomes emerged from the CoPs. First, a joint identity and understanding were created within and across the CoPs. Additionally, CO vocabulary was developed, which also served to differentiate such observatories from CS initiatives. Next, scientific papers and technical reports were cooperatively produced by CoP members that represent a synthesis of CoP member knowledge. The impacts of the WeObserve CoPs range from the uptake of jointly produced publications, novel cooperative CS projects, other new CoPs, joint grant proposals, and the integration of CS data into SDG monitoring.

Essential ingredients to the success of these CoPs included extensive stakeholder engagement and the CoPs being steered by the underpinning values of the CS community. The importance of the *community* in a CoP was reaffirmed and achieved by putting the members at the heart of its creation. The co-design method of the WeObserve approach ensures the CoPs are valuable for the members and fosters cooperative learning, sharing and development of resources and generating new perspectives. Furthermore, the WeObserve CoPs generated knowledge that can guide the formation of future communities of practice by modeling how practitioners can come together to meaningfully discuss, network, and advance endeavors of mutual interest.

The appetite and need for such CoPs is evident through the multiple new CoPs that have emerged since WeObserve ended. The online components of the WeObserve CoPs have also proved highly relevant for a post-COVID-19 world.

As this study shows, co-designed CoPs provide a space to learn and develop ideas in a safe and encouraging environment. This is particularly needed in the growing field of CS, where practitioners are often outnumbered in their organization. Furthermore, as CS becomes more recognized, the need for best practices will grow. Co-designed CoPs can contribute to establishing and sharing such best practices. This evaluation highlights the diverse and transformative potential of CoPs focused on CS.

## Supplementary information

Supplementary material

## Data Availability

No datasets were generated or analysed during the current study.

## Footnotes

1 https://weobserve.eu/ (2017-2021).

2 In some contexts, CoPs are also referred to as Working Groups. We use the term CoPs throughout this paper, but in principle, the aspects and dynamics analysed and discussed here also apply to Working Groups.

3 The term knowledge consolidation stems from the knowledge management literature, some of which focuses on how to integrate and turn into capabilities knowledge that is spread across organisational units (Cohen and Levingthal, 1990) and to capture knowledge for re-use (e.g. in repositories) and embedding it in organisational memory via adjusted or new practices (e.g. Davenport and Prusak, 1998). While it suggests a focus on knowledge codification (i.e. making tacit knowledge tangible), knowledge consolidation is complementary to the social interactions “in practice” (Wenger, 1998) that CoPs are focused on. Knowledge consolidation is particularly relevant to overcoming the dispersion of practice-based knowledge related to CS. Rather than inferring that this is about capturing the “right” knowledge, we use the term here in the context of CoPs that can provide a means to bringing dispersed as well as conflicting knowledge together and exploring diverse practices.

4 Across 18 questions for which comments were possible, there were 62 unique responses, 2-6 per question. The last two questions of the survey were open and received 20 and 16 answers, respectively.

5 Some respondents indicated “not able to judge” for some questions related to a particular CoP that they were not a member of, resulting in varying total numbers of responses per question per CoP.

6 WeObserve-CoP-Terms-of-Reference.pdf.

7 e.g., the GEO Citizen Science Activity and the Open Geospatial Consortium (OGC) Citizen Science Domain Working Group

8 https://www.weobserve.eu/cops-glossary/.

9 https://crops-cs.eu/.
