# Correction: Consolidating Dispersed Knowledge About Citizen Science and Citizen Observatories: Experiences from the Four WeObserve Communities of Practice

**DOI:** 10.1007/s00267-026-02548-4

**Published:** 2026-06-26

**Authors:** Uta Wehn, Dilek Fraisl, Joan Masó Pau, Mohammad Gharesifard, Linda See, Gerid Hager, Jessica L Oliver, Tova Crystal, Raquel Ajates, Ane Bilbao, Eglė Butkevičienė, Carlo Andrea Biraghi, Jillian Campbell, Laurent Durieux, Eugenio Gervasini, Margaret Gold, Gitte Kragh, Andreas Matheus, Stephen Mac Feely, Lukas Mocek, Maina Muniafu, Lea Shanley, Ali Yousefi

**Affiliations:** 1 CWTS - Centre for Science & Technology Studies, Leiden University, Leiden, The Netherlands; 2 IHE Delft Institute for Water Education, Delft, The Netherlands; 3 International Institute for Applied Systems Analysis (IIASA), Laxenburg, Austria; 4 CREAF, Barcelona, Spain; 5 University of Groningen, Groningen, The Netherlands; 6 Australian Citizen Science Association Inc. (ACSA), Maleny, QLD Australia; 7 Consejo Superior de Investigaciones Científicas, Madrid, Spain; 8 Biotechnology Department, Delft University of Technology, Delft, The Netherlands; 9 Kaunas University of Technology, Kaunas, Lithuania; 10 Politecnico di Milano, Milano, Italy; 11 Secretariat of the Convention on Biological Diversity, UNEP, Montreal, QC Canada; 12 French National Research Institute for Sustainable Development, Paris, France; 13 European Commission, Joint Research Centre, Ispra, Italy; 14 Aarhus University, Aarhus, Denmark; 15 Secure Dimensions GmbH, Munich, Germany; 16 OECD, Boulogne Billancourt, France; 17 Sensor.Community, Stuttgart, Germany; 18 United States International University-Africa, Nairobi, Kenya; 19 International Computer Science Institute, Berkeley, CA USA; 20 Isfahan University of Technology, Isfahan, Iran

Correction to: Environmental Management

10.1007/s00267-026-02442-z

In this article, the e-mail address of the corresponding author, Uta Wehn, was incorrect and should be u.w.c.wehn@cwts.leidenuniv.nl. The co-author's name, Raquel Ajates, was incorrectly written as Ajates.

The original article has been corrected.

