# Supplementary material for: Consolidating Dispersed Knowledge About Citizen Science and Citizen Observatories: Experiences from the Four WeObserve Communities of Practice
Source: Environ Manage. 2026 Apr 25;76(5):172. doi: 10.1007/s00267-026-02442-z (PMC13110208; doi:10.1007/s00267-026-02442-z)
Supplement: Supplementary file 1 — Supplementary material [file 267_2026_2442_MOESM1_ESM.docx]

**Supplementary material** for Wehn et al. (2026) Consolidating Dispersed Knowledge About Citizen Science and Citizen Observatories: Experiences from the Four WeObserve Communities of Practice, Environmental Management, https://doi.org/10.1007/s00267-026-02442-z

**Table 1 - Conceptual framework**

| **Aspect** | **Element** | **Sub-element** | | | **Questions** |
| --- | --- | --- | --- | --- | --- |
| **Inputs** | **Resources** | **Available resources** [Resources provided by WO and efforts by members] | Time resource | Time spent by members on CoP activities | - How long have you been a member of the CoP?** - How often are members involved in face-to-face CoP activities?** - How often are members involved in online CoP activity?** - On average, how many hours do you spend on the CoP(s) activities per month?* |
|  |  |  |  | Time spent by WO staff on organizing CoP activities | - How much time did it cost you per month to organize the CoP activities [Only for the CoP leads]* |
|  |  |  | Financial resources | Budget | - What was the source of funding for establishing and running the WO CoPs?** - How much funding was made available for establishing and running the WO CoPs?** - In what form(s) was the funding made available to the CoPs? (e.g. meeting space, online facilities, travel budget, technical/organizational support, etc)** |
|  |  |  |  | Financial resources spent from the members’ side | - What types of costs did you or your organization incur for your participation in the WeObserve CoP(s)?* |
| **Process** | **Participation and facilitation dynamics** | **Goals and objectives** [Main CoP objectives and members’ goals] | Overarching CoP objectives | The CoP objective | - What are the agreed-upon objectives of each CoP?** |
|  |  |  | Members' goals | | - What are your main expectations from participating in the WeObserve CoP(s)?* |
|  |  | **members** [Who participated in the CoPs?] | Geographic scope | | - What is the geographic distribution of the members of each CoP?** |
|  |  |  | (non)participant groups | Members | - Who participates in each CoP and in which role?** |
|  |  |  |  | Expertise of the members | - What is the background and expertise of the CoP members?** |
|  |  | **Community activities, interactions and networking**  [Details of community activities, interactions and networking opportunities] | Pattern of communication | Internal communications | - For you, what were the most important communication channels for communicating with the WeObserve CoP(s)? (Please indicate top 3 per CoP)* |
|  |  |  |  | External communications | - What were the most important communication channels for the WeObserve CoP(s) for reaching relevant external stakeholders? (please choose the top 3)* |
|  |  |  | Level of participation | | - What is your role in each of the WeObserve CoPs? (participant, observer, CoP chair)* |
|  |  |  | Level of activity | | - What were significant events? What happened?** |
|  |  |  | Level of engagement | | - How do you rate the quality of the engagement of members (e.g. intensity of discussions and contributions) in the WeObserve CoPs?* |
|  |  |  | Quality of interactions | | - How useful were your interactions with others in the WeObserve CoP(s)?* |
|  |  |  | Networking | | - Please indicate the extent of your agreement with the following statement: "The set-up of the WeObserve CoP(s) allows me to build or strengthen relationships and networks".* |
|  |  |  | Collaboration | | - Please indicate the extent of your agreement with the following statement: "The set-up of the WeObserve CoP(s) allows me to create collaboration opportunities with others".* |
|  |  | **Facilitation**  [Reflection on the facilitation process] | Activities | | - What activities were facilitated in each CoP?** |
|  |  |  | (Self)evaluation | | - How do you rate the facilitation of the WeObserve CoP(s) interactions during the regular online meetings?* |
| **Results** | **Achievement of goals and objectives** | **Level of achievement of goals and objectives**  [The extent to which the CoP objectives and members goals are achieved] | Perceptions about the level of achievement of CoP objectives | | - To what extent do you think the objectives of the WeObserve CoP(s) have been achieved so far?* |
|  |  |  | Perceptions about the level of achievement of members’ goals | | - To what extent have your expectations for participating in the WeObserve CoP(s) been met so far?* |
|  | **Individual/ organizational outcomes** | **Potential value: Knowledge capital**  [Activities and interactions can produce “knowledge capital” whose value lies in its potential to be realized later] | Personal assets (human capital) | | - How useful has your participation in the WeObserve CoP(s) been for you personally, e.g. for developing new skills or knowledge, change in understanding of a specific topic, finding out what others are doing on the topic, and/or getting inspired?* |
|  |  |  | Relationships and connections (social capital) | | - Please indicate the extent of your agreement with the following statement: "Participation in the WeObserve CoP(s) has created new collaboration opportunities for me".* |
|  |  |  | Resources (tangible capital) | | -To what extent has participation in the WeObserve CoP(s) enabled you to access (new) resources such as tools, methods, and processes?* |
|  |  |  | Collective intangible assets (reputational capital) | | - To what extent has the following been achieved: recognition of the collective expertise of the WeObserve CoP(s) by peers in the field of citizen science?* |
|  |  |  | Transformed ability to learn (learning capital) | | - To what extent has your participation in the WeObserve CoP(s) transformed HOW you learn?* |
|  |  | **Applied value: Changes in practice** [Looking at applied value means identifying the ways practice has changed in the process of leveraging knowledge capital] | Implementation of advice/solutions/insights | | - For you, to what extent have the insights, advice or solutions generated in the WeObserve CoP(s) been applicable so far?* |
|  |  |  | Innovation in practice | | - To what extent has participation in the WeObserve CoP(s) resulted in new ways of doing things, new perspectives and/or new concepts in the field of citizen science?* |
|  |  |  | Use of tools and documents to inform practice | | - To what extent have the outputs or methods produced by the WeObserve CoPs been used or applied, including outside the WeObserve CoPs?* |
|  |  |  | Use of social connections | | - To what extent have you leveraged the relationships /network(s) in the WeObserve CoP(s) so far?* |
|  | **Emerging impacts** | **Emerging impacts**  [Impacts that may materialize over a longer period because of the CoPs] | *Impacts (+/-, (un)intended)* | | - What are future impacts (positive/negative) resulting from the WeObserve CoP(s)?* |
|  | **Future developments** | **Future developments** [Points for improvement of the WO CoP(s) in the future] | *Future developments* | | - In future, which one or two things do you think we should add, change or improve when continuing the WeObserve CoPs or establishing new CoPs? Please provide your response in terms of generic aspects across the CoPs or for the specific CoP(s) that you participated in, as appropriate.* |


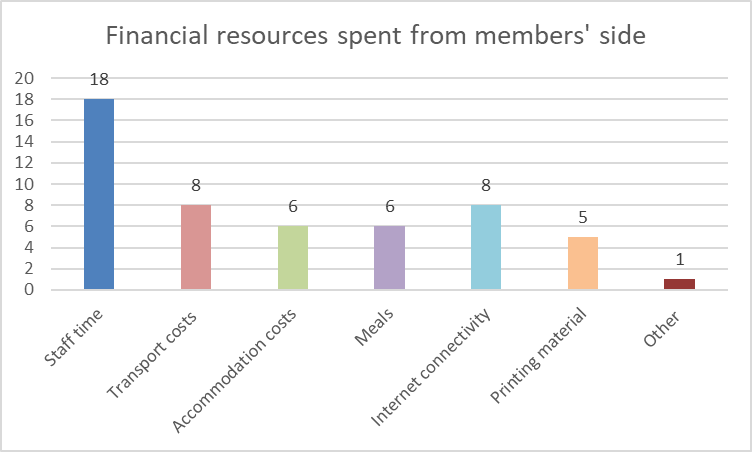


*N=28*

***Fig. 1*** *Financial resources spent from members side*


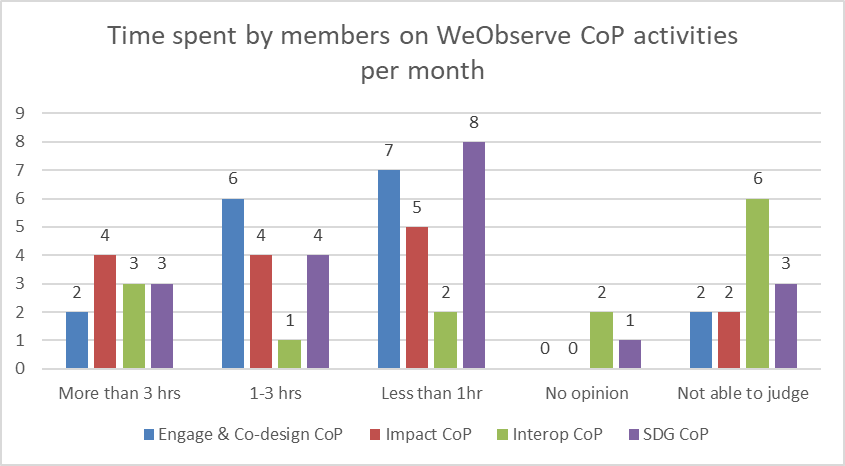


*n_1_=17; n_2_=15; n_3_=14; n_4_=19*

***Fig. 2*** *Time spent by CoP members on the WeObserve activities per month*


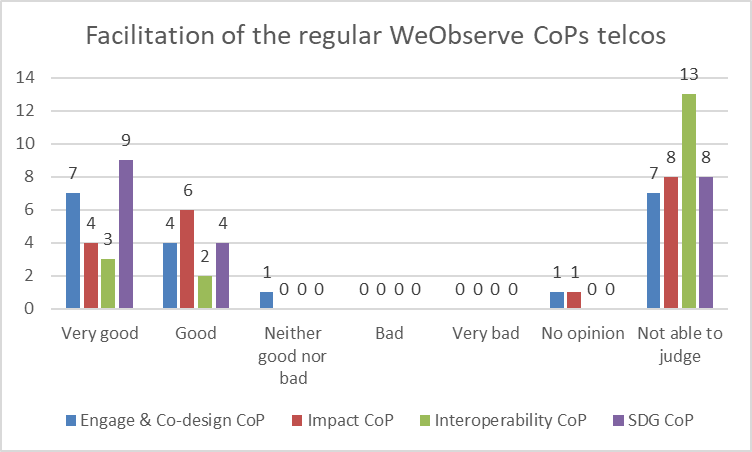


*n_1_ =20; n_2_ = 19 n_3_ = 18; n_4_ =21*

***Fig. 3*** *Survey responses regarding facilitation of the regular WeObserve CoPs online meetings*

**Table 2** Total number of members per CoP

| **CoP title** | **# of participants***  Total # (# external to the WeObserve project) | **# of observers** | **Total # of members per CoP**** |
| --- | --- | --- | --- |
| Engage & Co-design | 35 (19) | 132 | 167 |
| Impact | 25 (11) | 122 | 147 |
| Interoperability | 33 (24) | 102 | 135 |
| SDG | 50 (32) | 125 | 175 |

*participants are individuals who attended two or more of the CoP online meetings.

**175 people registered for more than one CoP


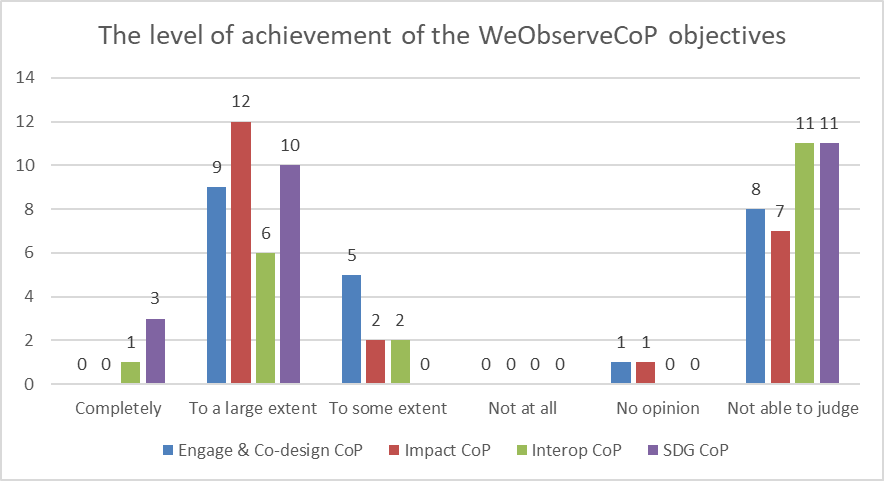


*n_1_ =23; n_2_ = 22; n_3_ = 19; n_4_ =24*

***Fig.4*** *Level of achievement of the WeObserve CoPs’ objectives*


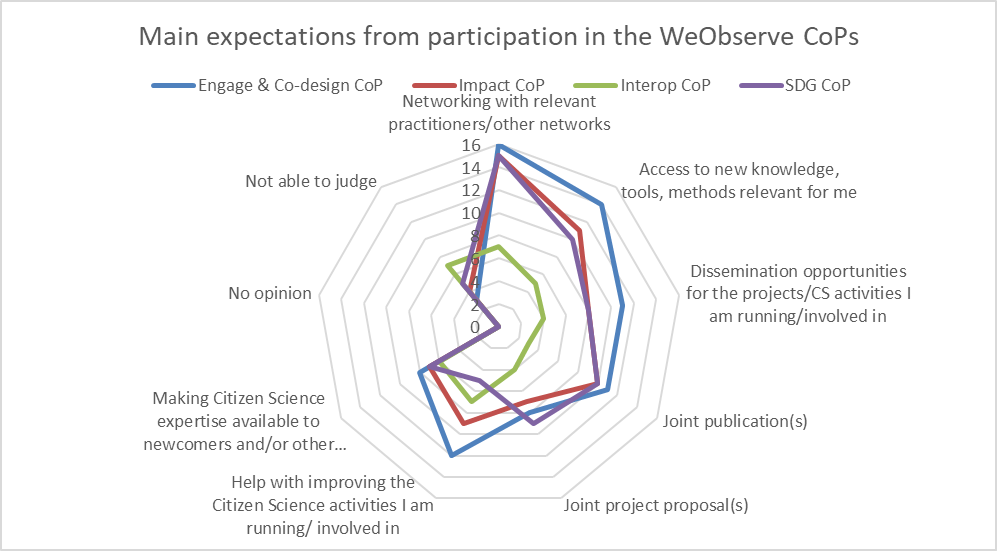


*n_1_ =21; n_2_ = 18; n_3_ = 10; n_4_ =21*

***Fig. 5*** *The main expectation from participation in the WeObserve CoPs*


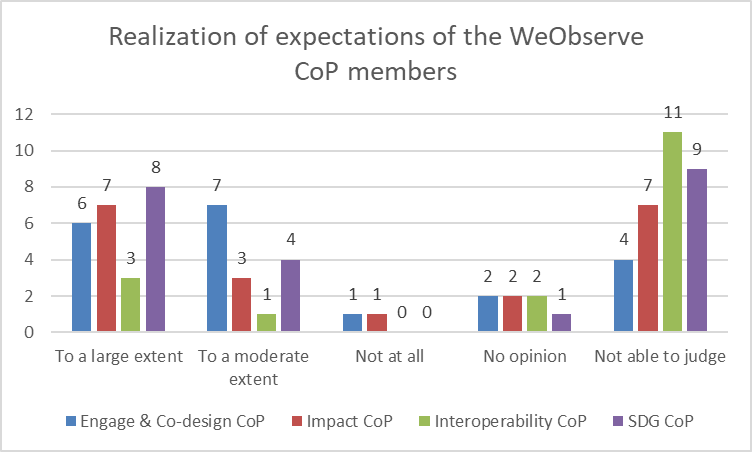


*n_1_ =20; n_2_ = 20; n_3_ = 17; n_4_ =22*

***Fig. 6*** *The realisation of expectations of the WeObserve CoP members*


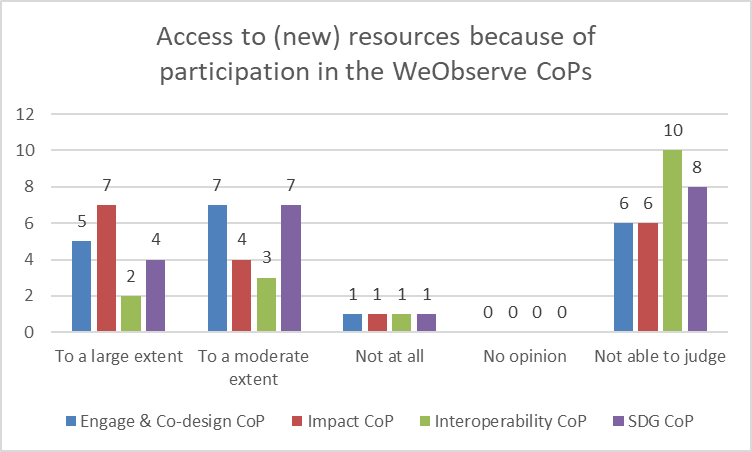


*n_1_ =20; n_2_ = 18; n_3_ = 16; n_4_ =20*

***Fig. 7*** *Access to (new) resources because of participation in the WeObserve CoPs*


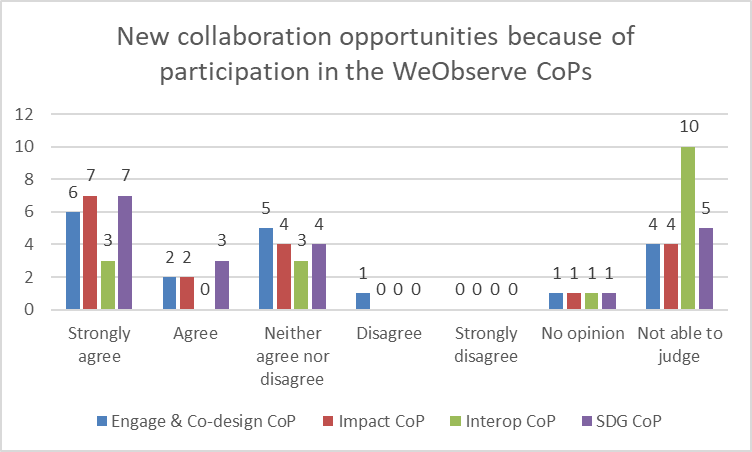


*n_1_ =19; n_2_ = 18; n_3_ = 17; n_4_ =20*

***Fig. 8*** *New collaboration opportunities because of participation in the WeObserve CoPs*


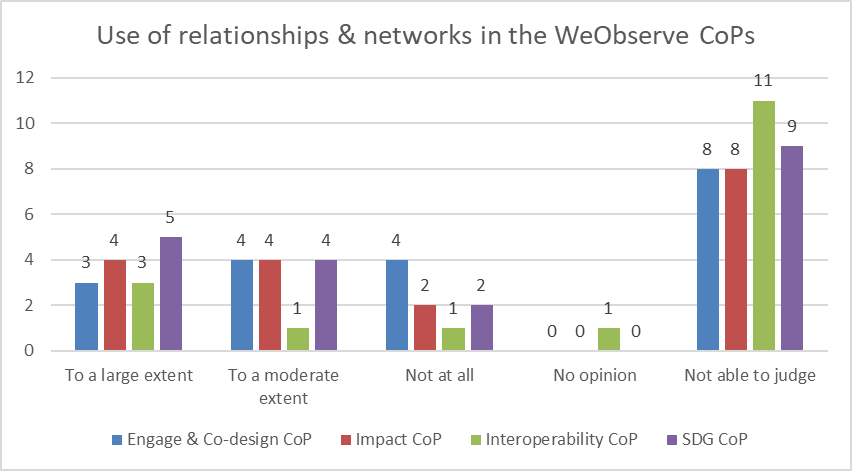


*n_1_ =19; n_2_ = 18; n_3_ = 17; n_4_ =20*

***Fig. 9*** *Use of relationships & networks in the WeObserve CoPs*


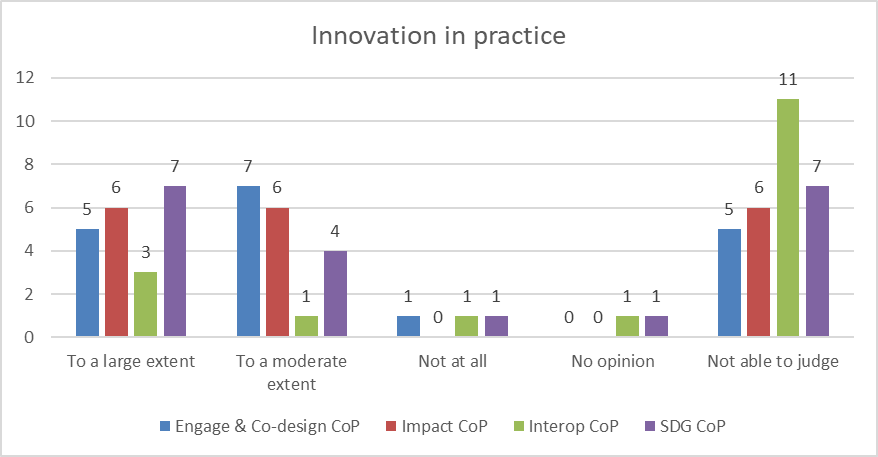


*n_1_ =18; n_2_ = 18; n_3_ = 17; n_4_ =20*

***Fig. 10*** *Innovation in practice*

***Table 3*** *New CoPs based on the WeObserve approach for CoPs*

| **CoP title** | **Domain** | **Start date, community** | **Activities** | **Results/ Impacts** |
| --- | --- | --- | --- | --- |
| Citizen Science & Open Science (CS & OS CoP),  established under the Citizen Science Global Partnership (CSGP) | Cooperation between CS & OS  Development and implementation of OS  Support for UNESCO to reflect the Citizen Science perspective on Open Science and its development in future. | July 2020  >200 members  Practitioners of CS & OS | - Wrote a short paper feeding into the process of developing the UNESCO OS Recommendation (Wehn et al., 2020)^1^ - Elaborated feedback on the first draft Recommendation - Participated as an observer of the CS&OS CoP co-chair in the formal Recommendation negotiation process & official statement - Developed a technical report on open engagement of societal actors in OS Wehn and Hepburn, (2022)^2^ - Created a guidance document on open engagement of societal actors for the UNESCO OS Toolkit - Wrote an academic paper with the scholarly underpinnings of the guidance document (Wehn et al., 2024)^3^ - Contribution to the first UNESCO OS Outlook | Two pillars in UNESCO recommendations on OS are related to citizen science and knowledge production with societal stakeholders  1) open engagement of societal actors,  2) open dialogues with other knowledge systems  Continued collaboration with UNESCO on key aspects of implementing the UNESCO Recommendation on OS |
| Marine Citizen Science CoP, University of Gothenburg | Marine citizen science as a new topic cutting across faculties and disciplines at the University of Gothenburg | October 2021 - May 2022,  University staff across 4 faculties | 4 dedicated workshops (half a day each) on the following co-designed topics:   - *Citizen Science terminology & typology* - *Citizen and stakeholder engagement* - *Citizen Science and data quality* - *Measuring impacts of citizen science*   One full-day mini-conference, incl. an excursion on the marine research vessel of the University of Gothenburg. | Faculty members across a range of disciplines connected and engaged with CS concepts and practices |
| Urban ReLeaf CoP | Promotes and co-develops knowledge on inclusive urban green transitions.  Targets city representatives, relevant stakeholders, academics, civil society organisations (CSOs) and related communities | October 20234  >70 members  WG 1 - Local government representatives, Citizen associations, CSOs, Business community.  WG 2 - Key stakeholders: Municipalities, Authoritative data agencies, GEO, EuroGEO, Copernicus, EEA, ECSA  WG 3 - Key stakeholders: Municipalities, Urban Planners, Architects, EPAs. | Three Working Groups (WG):  -WG 1: Triggering innovation in public authorities with citizen-powered science;-WG 2: Coupling citizen science and EO for European Green Deal & SDG 11 monitoring; -WG 3: Urban design foresight for Nature-Based Solutions and Blue-Green Infrastructure scenario planning; Key Stakeholders: Municipalities, Urban Planners, Architects, EPAs  Objectives and activities of the WG are determined through a co-design process.  Cross-WG activities included, e.g., Urban Innovation Rallye, an interactive game about urban green transformations (introduced at ECSA conference 2024). | WG 1:  Best practices on how to successfully use citizen science data for policy monitoring/revision and adoption.  WG 2:  - Pending publication on the role of citizen science data for the Global Urban Monitoring Framework from UN-HABITAT.  - Contributions to the Collaborative on Citizen Data (UN Statistical Division).  WG 3:  Pending publication on community urban design foresight for NBS and BGI scenario planning |
| CROPS (Curating, Replicating, Orchestrating, and Propagating Citizen Science across Europe) Transnational Communities | Establishing innovative, inclusive and representative mechanisms and associated communities and societal coalitions for the upscaling of citizen science activities. | mid-2025 | Objectives and activities are to be determined following a co-design process. | N/A |
| CitiObs Alliance & Fellows CoP | EU-funded project enhancing Citizen Observatories for healthy, sustainable, resilient and inclusive cities.  Targets community-based groups, civil society organisations, city and provincial policymakers and relevant stakeholders. | December 2024 | Objectives and activities are to be determined following a co-design process based on the WeObserve CoP model. Sub-groups or Working Groups will be established based on the identified needs of the participating Citizen Observatories, covering such subjects as inclusive outreach and engagement and policy impact. | N/A |
| more4nature (Empowering citizens in collaborative environmental compliance assurance via MOnitoring, REporting and action) CoPs | Establishing inclusive CoPs focused on peer learning within thematic topics, across topics, between authorities and citizen science initiatives involved in 40 cases related to citizen science and environmental compliance assurance | 2025 | Five CoPs:  CoP 1: Enabling public authorities to integrate citizen-generated data in ECA; Key stakeholders: local, regional, national, EU authorities.  CoP 2: Citizen science initiatives for ECA; Key stakeholders: Citizen science initiatives, research performing organisations, NGOs.  CoP 3: Biodiversity Citizen Science Initiatives for ECA; Key stakeholders: Citizen science initiatives, research performing organisations, NGOs, authorities, policy makers.  CoP 4: Zero Pollution Citizen Science Initiatives for ECA; Key stakeholders: Citizen science initiatives, research performing organisations, NGOs, authorities, policy makers.  CoP 5: Deforestation Prevention Citizen Science Initiatives for ECA; Key stakeholders: Citizen science initiatives, research performing organisations, NGOs, authorities, policy makers. | N/A |

**Table 4:**  *A summary of CoP-produced publications with respective citation counts as a measure of uptake (citation count values as of 10/04/2026)*

| **#** | **Title, authors & DOI** | **Publication date** | **WeObserve CoP** | **Citations from Scopus** | **Citations from Google Scholar** |
| --- | --- | --- | --- | --- | --- |
| 1 | Citizen science and the United Nations Sustainable Development Goals [10.1038/s41893-019-0390-3](http://dx.doi.org/10.1038/s41893-019-0390-3) | 2019 | SDG CoP | 498 | 751 |
| 2 | Citizen science and the data integration for understanding marine litter  <https://pure.iiasa.ac.at/id/eprint/16095/?template=default_internal> | 2019 | SDG CoP | *Not indexed* | 16 |
| 3 | Counting on the World to Act: A Roadmap for Governments to Achieve Modern Data Systems for Sustainable Development <https://countingontheworld.sdsntrends.org/> | 2019 | SDG CoP | *Not indexed* | 4 |
| 4 | The role of combining national official statistics with global monitoring to close the data gaps in the environmental SDGs  [10.3233/SJI-200648](https://content.iospress.com/articles/statistical-journal-of-the-iaos/sji200648) | 2020 | SDG CoP | 27 | 37 |
| 5 | Mapping citizen science contributions to the UN sustainable development goals  <https://doi.org/10.1007/s11625-020-00833-7> | 2020 | SDG CoP | 315 | 516 |
| 6 | The value of citizen science for flood risk reduction: Cost-benefit analysis of a citizen observatory in the Brenta-Bacchiglione catchment, Hydrology and Earth System Sciences, 24, 5781-5798,<https://doi.org/10.5194/hess-24-5781-2020>. | 2020 | Impact CoP | 30 | 55 |
| 7 | OGC Citizen Science Interoperability Experiment Engineering Report, <https://docs.ogc.org/per/19-083.html> | 2020 | Interop CoP | *Not indexed* | *1* |
| 8 | UNEP Report: Measuring Progress: Environment and the SDGs <https://www.unep.org/resources/publication/measuring-progress-environment-and-sdgs> | 2021 | SDG CoP | *Not indexed* | *Not Indexed* |
| 9 | Capturing and communicating the impact of citizen science for policy: A storytelling approach, <https://doi.org/10.1016/j.jenvman.2021.113082>. | 2021 | Impact CoP | Not indexed | 39 |
| 10 | Impact Assessment of citizen science: state of the art and guiding principles for a consolidated approach  <https://doi.org/10.1007/s11625-021-00959-2> | 2021 | Impact CoP | 79 | 119 |
| 11 | Demonstrating the potential of Picture Pile as a citizen science tool for SDG monitoring [10.1016/j.envsci.2021.10.034](https://doi.org/10.1016/j.envsci.2021.10.034). | 2022 | SDG CoP | 33 | 47 |
| 12 | OGC Best Practice for using SensorThings API with Citizen Science~~,~~ <https://docs.ogc.org/bp/21-068.pdf> | 2022 | Interop CoP | *Not indexed* | *Not indexed* |
| 13 | The contributions of citizen science to SDG monitoring and reporting on marine plastics <https://link.springer.com/article/10.1007/s11625-023-01402-4> | 2023 | SDG CoP | 24 | 32 |
| 14 | Citizen science for monitoring the health and well-being related to Sustainable Development Goals and the World Health Organization’s Triple Billion Targets | 2023 | SDG CoP | 19 | 30 |
| 15 | The Third Global session of the UNEP Science Policy Business Forum (SPBF) - High-level opening session talk by the SDG cop chair presenting the cop mapping paper  <https://un-spbf.org/event/un-spbf-integrated-solution-fornature/> | 2021 | SDG CoP | *Not indexed* | *Not indexed* |

1 Wehn, U., Goebel, C., Bowser, A., Hepburn, L. & Haklay, M. (2020) Global Citizen Science perspectives on Open Science. Short Paper for UNESCO Advisory Body on the Open Science Recommendation, May

2 Wehn, U. and Hepburn, L. (eds) (2022) Guidance for the implementation of the UNESCO Open Science Recommendation regarding "Opening science to society", Report for the UNESCO Toolkit on Open Science, Citizen Science & Open Science Community of Practice, November, DOI: <https://zenodo.org/record/7472827>

3 Wehn, U., Ajates, R., Mandeville, C., Somerwill, L., Kragh, G., Haklay, M., (2024) Opening science to society: how to progress societal engagement into (open) science policies, Royal Society Open Science, 11:231309, https://doi.org/10.1098/rsos.231309
